# Circulating Plasma Adiposomes as a Partial Lipidomic Readout of Matched Subcutaneous Adipose Tissue: Implications for Interorgan Communication

**DOI:** 10.1002/cph4.70296

**Published:** 2026-11-01

**Authors:** Mohamed Saad Rakab, Imaduddin Mirza, Elsayed Metwally, Mohammed Morsy, Carlos Martinez, Mario Masrur, Chandra Hassan, Francesco M. Bianco, Brian T. Layden, Abeer M. Mahmoud

**Affiliations:** ^1^ Division of Endocrinology, Diabetes, and Metabolism, Department of Medicine, College of Medicine University of Illinois Chicago Chicago Illinois USA; ^2^ Department of Cytology and Histology, Faculty of Veterinary Medicine Suez Canal University Ismailia Egypt; ^3^ Research Informatics Core University of Illinois Chicago Chicago Illinois USA; ^4^ Department of Surgery, College of Medicine University of Illinois Chicago Chicago Illinois USA; ^5^ Jesse Brown Veterans Affairs Medical Center Chicago Illinois USA; ^6^ Department of Kinesiology and Nutrition, College of Applied Health Sciences University of Illinois Chicago Chicago Illinois USA; ^7^ Department of Pathology, South Egypt Cancer Institute Assiut University Assiut Egypt

**Keywords:** adipose tissue, adiposomes, cardiometabolic disease, circulating biomarkers, concordance, extracellular vesicles, interorgan communication, lipidomics, obesity

## Abstract

**Background:**

Prior studies established adipose‐derived extracellular vesicles, or adiposomes, as mediators of interorgan communication with lipidomic signatures linked to cardiometabolic health. Because adiposomes signal to distant organs via the circulation, we examined whether circulating adiposome lipidomes reflect matched subcutaneous adipose tissue adiposomes.

**Methods:**

This cross‐sectional paired lipidomic study included 22 subjects with matched circulating and subcutaneous adipose tissue adiposome samples. Untargeted lipidomics quantified 773 lipid features across 18 detected broad classes. Spearman correlation was the primary across‐subject species‐level metric; pooled and within‐subject Pearson and Spearman correlations, principal component analysis, Bland–Altman analysis, class composition, and matched‐versus‐mismatched similarity were complementary measures. Additional analyses evaluated sex, cardiometabolic phenotypes, and focused species‐clinical associations.

**Results:**

Across 770 species with valid across‐subject estimates, the median plasma‐subcutaneous Spearman *ρ* was 0.101 and no species survived Benjamini‐Hochberg false‐discovery‐rate correction, although 553 (71.8%) correlations were positive. Mean broad‐class composition was highly conserved (Spearman *ρ* = 0.996; Pearson *r* on log_2_ percentages = 0.996). Matched whole‐profile similarity was not greater than mismatched similarity (median *ρ* = 0.818 vs. 0.811; *p* = 0.620), and the matched sample was the closest pair for 0 of 22 participants. Neither sex nor any prespecified cardiometabolic phenotype explained matched‐profile similarity after correction, and no focused species‐clinical association survived full‐screen FDR correction.

**Conclusions:**

Circulating and subcutaneous adiposome lipidomes share a highly conserved mean class architecture, but individual species, subject‐specific matching, and clinical‐association patterns are not interchangeable. Plasma adiposomes may therefore serve as complementary circulating readouts of adipose‐derived interorgan lipid signaling rather than direct tissue substitutes.

## Graphical abstract

In 22 participants, circulating plasma adiposome lipidomes showed moderate global concordance with matched subcutaneous adipose tissue adiposomes across 773 lipid features and 18 classes. These findings support plasma adiposomes as accessible, non‐invasive biomarkers of adipose lipid biology while recognizing compartment‐specific differences.

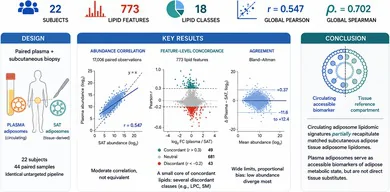

## 1 Introduction

Adipose tissue is increasingly recognized as an active endocrine and immunometabolic organ rather than a passive depot for energy storage (Rakab et al. 2025). Beyond secreting adipokines and inflammatory mediators, adipose tissue communicates locally and systemically through extracellular vesicles, which we refer to as adiposomes (Mirza et al. 2023). These vesicles are now viewed as important components of the adipose secretome, contributing to autocrine and paracrine signaling within adipose tissue and endocrine communication with distant metabolic organs (Le Lay and Scherer 2025). These adiposomes transfer proteins, nucleic acids, metabolites, and lipids between cells, allowing adipose tissue to influence insulin sensitivity, inflammation, vascular function, and whole‐body metabolic homeostasis (Malaguarnera et al. 2025). This signaling capacity positions adiposomes as mediators of interorgan crosstalk between adipose tissue and distant targets such as the liver, skeletal muscle, and pancreatic β‐cells, where they can shape hepatic lipid handling, muscle glucose uptake, and insulin secretion. As a result, they have drawn attention as a link between dysfunctional adipose tissue and the cardiometabolic complications that accompany it.

The lipid cargo of adiposomes is biologically relevant in obesity and cardiometabolic disease, conditions characterized by altered lipid handling, adipose inflammation, insulin resistance, and endothelial dysfunction (Rakab et al. 2025; Mirza et al. 2023; Mahmoud et al. 2025). Using adipose tissue biopsies, prior studies established the molecular cargo of adipose tissue‐derived adiposomes as mechanistic mediators of vascular dysfunction and predictive biomarkers of inflammation, insulin resistance, and metabolic disease (Rakab et al. 2025; Mirza et al. 2023; Mahmoud et al. 2025; Metwally et al. 2025). In a cohort of 75 obese participants, lipidomics profiling of adiposomes showed enrichment in ceramides, FFAs, acylcarnitines, and inflammatory lipids, and depletion of vasoprotective lipid species like fatty acyl esters of hydroxy fatty acids (FAHFAs) and phosphocholines (Mahmoud et al. 2025; Hussein et al. 2024). These patterns correlated with insulin resistance, systemic inflammation, impaired nitric oxide (NO) bioavailability, and reduced arteriolar vasodilation and predicted Type 2 diabetes (T2D) and hypertension with > 85% accuracy, supporting their translational potential as biomarkers of cardiometabolic diseases (Rakab et al. 2025; Mirza et al. 2023). Following bariatric surgery, we observed reductions in the adiposome cargo of inflammatory and lipotoxic lipids along with increases in potentially vasculoprotective lipid cargo such as FAHFAs (Metwally et al. 2026). These changes mirrored post‐bariatric improvements in BMI, vasoreactivity, insulin sensitivity, and systemic inflammation. We further established adipose tissue‐derived adiposomes as mechanistic contributors to vascular cell function (Mirza et al. 2023; Metwally et al. 2025). Adiposomes from obese diabetic adults disrupted endothelial caveolae, causing endothelial nitric oxide synthase (eNOS) uncoupling and oxidative stress; effects partially prevented by glycosphingolipid cargo depletion (Mirza et al. 2023). In smooth muscle cells, these adiposomes dysregulated intracellular Ca^2+^ signaling, impaired the ATP‐dependent potassium channel activity, and promoted hypercontractility; effects reversed by ceramide cargo depletion (Metwally et al. 2025).

It is worth noting that prior work has relied primarily on adiposomes obtained from invasive fat biopsies, limiting the clinical applicability of this research in risk stratification and therapeutic monitoring in population‐level studies. To date, it remains unknown whether lipidomic profiles are recapitulated in circulating adiposomes; if confirmed, this would clearly establish a minimally invasive approach for investigating adipose tissue remodeling that impacts metabolic and vascular function. This question is also central to interorgan communication. Because adiposomes signal to distant organs via the circulation, the circulating adiposome lipidome represents the adipose‐derived lipid signal that is systemically available to target tissues such as the liver, skeletal muscle, and vasculature. In the current cross‐sectional paired lipidomics study, we compared circulating adiposomes with matched subcutaneous adipose tissue adiposomes from 22 subjects using an untargeted lipidomic pipeline that quantified 773 lipid features across 18 lipid classes. The analysis evaluated global, compositional, subject level, feature level, lipid class specific, multivariate, and agreement‐based concordance between compartments, together with the cross‐compartment agreement of lipid–clinical associations.

## 2 Methods

### 2.1 Subject Recruitment

Adults with obesity (*n* = 22; 19 women and 3 men; 11 Black, 9 Hispanic, and 2 White participants) were recruited through the UIC bariatric clinics. The sex distribution reflected the available bariatric surgery cohort rather than sex‐specific eligibility criteria. Inclusion criteria included age 18 years or older and scheduled sleeve gastrectomy. Exclusion criteria included familial hyperlipidemia, cardiovascular, cerebrovascular, renal, thyroid disease, cancer, current tobacco use, or another metabolic disease. Participants using lipid‐modifying supplements or medications, including statins and fibrates, were excluded to reduce drug‐related effects on adiposome lipid profiles. Eligible participants provided written informed consent and attended a study visit within 1 week before surgery for plasma collection and anthropometric measurements; subcutaneous adipose tissue was collected on the day of surgery. The protocol conformed to the Declaration of Helsinki (World Medical Association 2013) and was approved by the University of Illinois Chicago Institutional Review Board (protocol #2021‐1113; approval date October 25, 2021).

### 2.2 Adiposome Isolation

Subcutaneous adipose tissue (SAT) biopsies were obtained during bariatric surgeries (~400 mg each). Fat biopsies were prepared using the methods we published (Mirza et al. 2023). Briefly, adipose tissue was rinsed with sterile Medium 199 (Gibco, Waltham, MA, USA), minced, and digested with Type I collagenase (Worthington) and 4% bovine serum albumin (BSA) in Medium 199. Following filtration and centrifugation (500 *g*), floating mature adipocytes were collected and cultured on inserts in Medium 199 supplemented with 1% penicillin/streptomycin and 5% exosome‐free fetal bovine serum (FBS). After 24–48 h, conditioned media underwent sequential centrifugation (1000 *g* for 5 min, 15,000 *g* for 15 min), filtration (0.45 μm), and ultracentrifugation at 150,000 *g* for 2 h to isolate adiposomes. Pellets were resuspended and characterized using nanoparticle tracking analysis (NanoSight NS300, Malvern Instruments Ltd., Malvern, UK).

Circulating adiposomes were isolated from ~20–25 mL of plasma (40–50 mL of blood) by centrifugation at 1200 *g* for 15 min and 15,000 *g* for 15 min to remove cells and debris, yielding platelet‐free plasma. The resulting supernatant was filtered through a 0.45 mm syringe‐driven filter, then ultracentrifuged at 150,000 *g* for 2 h. Pelleted adiposomes were resuspended in filtered PBS, followed by sequential solid‐phase immunodepletion of the most abundant non‐adipose EVs using antibodies against platelets (CD41), leukocytes (CD11b), endothelial cells (CD144), and erythrocytes (CD235a), following methods published by Connolly et al. (2018). In the current study, we further enriched the adiposome fraction by selective immunoprecipitation of adipocytic proteins using magnetic beads labeled with membrane‐associated adiponectin antibody (Welsh et al. 2024).

Adiposome proteins were extracted with RIPA buffer, quantified by BCA assay, separated on 4%–12% Bis‐Tris gels, and transferred to PVDF membranes. Membranes were incubated with primary antibodies against CD9, CD81, CD63, PPARγ, adiponectin, APOB, and FABP4 (Connolly et al. 2018; Welsh et al. 2024; Eguchi et al. 2016) (Cell Signaling, Danvers, MA, USA), followed by infrared secondary antibodies (LI‐COR Biosciences), and visualized using the Odyssey CLx infrared imaging system.

### 2.3 Adiposome Lipid Extraction and Analysis

Lipids were extracted from adiposomes via the Folch method (Folch et al. 1957) and analyzed on an Agilent 6545 Q‐TOF LC–MS system using a 2.1 × 100 mm Agilent Poroshell C18 (2.7 μm) column (Agilent Technologies, Santa Clara, CA, USA) at a flow rate of 300 μL/min (Köfeler et al. 2021). The gradient started at 70% solvent B (0–1 min), ramped to 86% B (3.5–10 min), reached 100% B (11–17 min), and was re‐equilibrated for 5 min. Key settings included 3000 V VCap, 145 V fragmentor, 12 L/min sheath gas at 350 degrees C, and 11 L/min drying gas at 200 degrees C. Lipid identification used Lipid Annotator software (Agilent), which generated an MS/MS library based on m/z precursors and retention times for each identified lipid (Koelmel et al. 2020). Profinder (vB.10.00, Agilent) extracted molecular features for peaks of at least 5000 counts with a quality score greater than 60 and at least two isotopes. Retention times were aligned to ±0.1 min within 5.0 ppm mass accuracy, and peaks were integrated using the Agile integrator. Plasma and adipose tissue EVs were isolated from standardized input amounts of plasma and tissue, respectively, and EV abundance was normalized to protein content (μg) rather than particle number before lipidomic analysis. LC–MS lipidomic signals were subsequently baseline‐corrected and normalized to internal‐standard signal, yielding internally standardized relative intensities. Concordance analyses were therefore based on these protein‐normalized, internally standardized values rather than absolute quantities per plasma volume, tissue mass, adipocyte number, EV particle number, or total lipid signal. Percentage‐of‐total‐signal normalization was applied separately and only for the lipid‐class composition analysis.

### 2.4 Statistical Analysis

This cross‐sectional study compared the lipidomic profiles of circulating plasma adiposomes with adiposomes isolated from matched subcutaneous adipose tissue in 22 subjects, each contributing paired samples processed under identical untargeted lipidomic pipelines yielding 773 lipid features. Features with greater than 20% missing values were excluded and remaining missing entries were imputed using half‐minimum imputation. Raw abundances were log_2_‐transformed to stabilize variance, with additional Pareto scaling applied for multivariate analyses. Each lipid feature was annotated with its parent class according to LIPID MAPS shorthand notation (Liebisch et al. 2020).

Across‐subject concordance of each lipid species was assessed primarily using Spearman *ρ* between matched plasma and subcutaneous adiposome abundances across 22 participants because the distributions were skewed and rank preservation was the main biological question. Pearson *r* on log_2_‐transformed abundances was used as a complementary measure of linear association, while Bland–Altman analysis was used separately to assess quantitative agreement beyond correlation. Species‐level *p* values were adjusted by the Benjamini‐Hochberg procedure, and the proportion of positive *ρ* values was compared with the 50% null expectation by exact binomial test. Global concordance was also assessed by pooling all 773 features across 22 participants into paired vectors (*n* = 17,006 observations) and computing Pearson *r* and Spearman *ρ*, with 95% confidence intervals derived by Fisher *z*‐transformation and bootstrap resampling (5000 iterations), respectively. Per‐subject concordance was evaluated across each matched 773‐element lipid profile, with per‐subject Pearson *r* computed on untransformed intensities. For continuity with the prespecified exploratory analysis, species were also described by Pearson thresholds as concordant (*r* > 0.3), discordant (*r* < −0.2), or neutral, and log_2_ fold changes were computed.

Lipid class enrichment among concordant versus discordant features was tested using Fisher's exact test with Benjamini–Hochberg correction (Benjamini and Hochberg 1995). Paired Wilcoxon signed‐rank tests assessed differential abundance for each feature between compartments. Principal component analysis was performed on the Pareto‐scaled matrix to visualize unsupervised compartment separation, with significance assessed by permutation testing (1000 permutations). Bland–Altman analysis evaluated agreement beyond correlation by computing the mean bias and 95% limits of agreement globally and by lipid class, with proportional bias tested via linear regression (Bland and Altman 1986). Sensitivity analyses included outlier exclusion and varying concordance classification thresholds across strict, default, and lenient criteria.

Lipid class composition was summarized as the mean percentage of total lipid signal contributed by each broad class in each compartment, and concordance of the composition profile was quantified by Spearman correlation and Pearson correlation on log_2_‐transformed class percentages. Class‐level clinical associations were estimated as Spearman correlations separately for plasma and subcutaneous compartments and corrected within compartment by the Benjamini‐Hochberg method. Cross‐compartment agreement of clinical effects was assessed at the species level by correlating plasma and subcutaneous effect‐size profiles and calculating sign concordance. Subject specificity was evaluated by comparing each participant's matched whole‐profile Spearman similarity with all mismatched plasma‐subcutaneous similarities. To test whether sex or cardiometabolic phenotype influenced this principal matched‐profile result, matched‐profile *ρ* was related to nine dichotomous phenotypes by rank‐biserial effect with 50,000‐label permutation testing and to 23 continuous variables by Spearman *ρ* with 20,000‐label permutation testing; Benjamini‐Hochberg correction was applied within the dichotomous and continuous families. The focused clinical matrix prespecified the ten most positively coupled species plus diacylglycerol (DG) 18:1_20:4 and acylcarnitine (ACar) 26:0 against nine continuous outcomes; its *q* values derive from the full 773‐species screen within clinical variable and compartment.

## 3 Results

The cohort included 19 women and 3 men and was racially diverse, with severe obesity and a high burden of metabolic, hepatic, inflammatory, and vascular abnormalities (Table 1). Median BMI was 43.5 kg/m^2^, and common clinical comorbidities included hypertension in 50.0% of participants, diabetes in 36.4%, dyslipidemia in 54.5%, and hepatic steatosis in 72.7%. Vascular dysfunction was also frequent, with impaired brachial flow‐mediated dilation (FMD) observed in 81.8% and impaired arteriolar FID in all participants.

**TABLE 1:** Baseline clinical characteristics of the study cohort.

Characteristic	Overall (*N* = 22)
Sex	
Female	19 (86.4)
Male	3 (13.6)
Race	
Black	11 (50.0)
Hispanic	9 (40.9)
White	2 (9.1)
Age, years	36 (30–43)
BMI, kg/m^2^	43.5 (41.8–46.6)
Total fat, %	49.1 (46.2–52.5)
Adiposity and cardiovascular measures	
Visceral fat mass, kg	1.91 (1.65–2.35)
Heart rate, beats/min	80 (73–94)
Systolic BP, mmHg	138 (124–146)
Diastolic BP, mmHg	81 (71–87)
Metabolic and lipid profile	
Fasting insulin, μIU/mL	14.3 (12.0–20.5)
Fasting glucose, mg/dL	96 (85–107)
HOMA‐IR	4.1 (2.9–5.1)
HbA1c, %	5.3 (5.1–5.6)
Total cholesterol, mg/dL	168 (154–194)
LDL, mg/dL	98 (80–117)
HDL, mg/dL	48 (40–55)
Triglycerides, mg/dL	124 (96–196)
Liver, vascular, and inflammatory markers	
Nitric oxide, μmol/L	3.18 (2.31–5.04)
Total bilirubin, mg/dL	0.4 (0.3–0.6)
Alkaline phosphatase, U/L	78 (67–95)
AST, U/L	15 (12–20)
ALT, U/L	16 (12–24)
Total protein, g/dL	7.0 (6.9–7.3)
Albumin, g/dL	4.2 (3.9–4.5)
Hemoglobin, g/dL	12 (11–13)
Brachial FMD, %	5.0 (3.8–6.2)
Arteriolar FID, %	48.3 (47.7–49.3)
Leptin, ng/mL	28.0 (23.4–38.8)
Adiponectin, μg/mL	4.8 (4.0–7.0)
Leptin/adiponectin ratio	5.3 (3.8–7.3)
IL‐6, pg/mL	26.7 (16.4–35.1)
CRP, mg/dL	4.0 (3.1–5.4)
Clinical classifications	
Hypertensive	11 (50.0)
Diabetic	8 (36.4)
Insulin resistant	9 (40.9)
Dyslipidemic	12 (54.5)
Hepatic steatosis	16 (72.7)
Impaired FMD	18 (81.8)
Impaired FID	22 (100.0)
Systemic inflammation	12 (54.5)
Impaired vascular function	15 (68.2)

*Note:* Values are median (IQR) for continuous variables and *n* (%) for categorical variables.

A total of 773 lipid features were quantified in adiposome samples obtained from 22 subjects, each contributing matched subcutaneous adipose tissue and plasma samples (44 total samples: 22 subcutaneous adipose tissue adiposome samples and 22 plasma adiposome samples). All 773 features passed quality control filtering (none exceeded the 20% missing value threshold). The lipid features spanned 18 detectable distinct lipid classes out of 19 prespecified classes based on similar previous studies (Mahmoud et al. 2025; Metwally et al. 2026). The most abundant classes were triacylglycerols (TG; *n* = 162), phosphatidylcholines (PC; *n* = 147), sphingomyelins (SM; *n* = 95), broad ceramides (Cer; *n* = 92), ether‐linked phosphatidylcholines (EtherPC; *n* = 40), phosphatidylethanolamines (PE; *n* = 35), fatty acids (FA; *n* = 29), lysophosphatidylcholines (LPC; *n* = 28), ether‐linked phosphatidylethanolamines (EtherPE; *n* = 26), phosphatidylserines (PS; *n* = 25), and diacylglycerols (DG; *n* = 22).

### 3.1 Global Concordance Between Compartments

Across all 17,006 paired observations (773 features × 22 subjects), plasma adiposome abundance showed a moderate positive correlation with subcutaneous adipose tissue adiposome abundance (Pearson *r* = 0.547, 95% CI: 0.536–0.557, *p* < 0.001; Figure 1). Spearman's rank correlation was substantially higher (*ρ* = 0.702, 95% bootstrap CI: 0.693–0.711, *p* < 0.001), suggesting that the rank ordering of lipid species is more faithfully conserved between compartments than the precise quantitative abundances. The difference between Pearson and Spearman coefficients (0.155) indicates that the linear relationship is attenuated by a subset of features with disproportionately large or small abundance differences.

**FIGURE 1:**
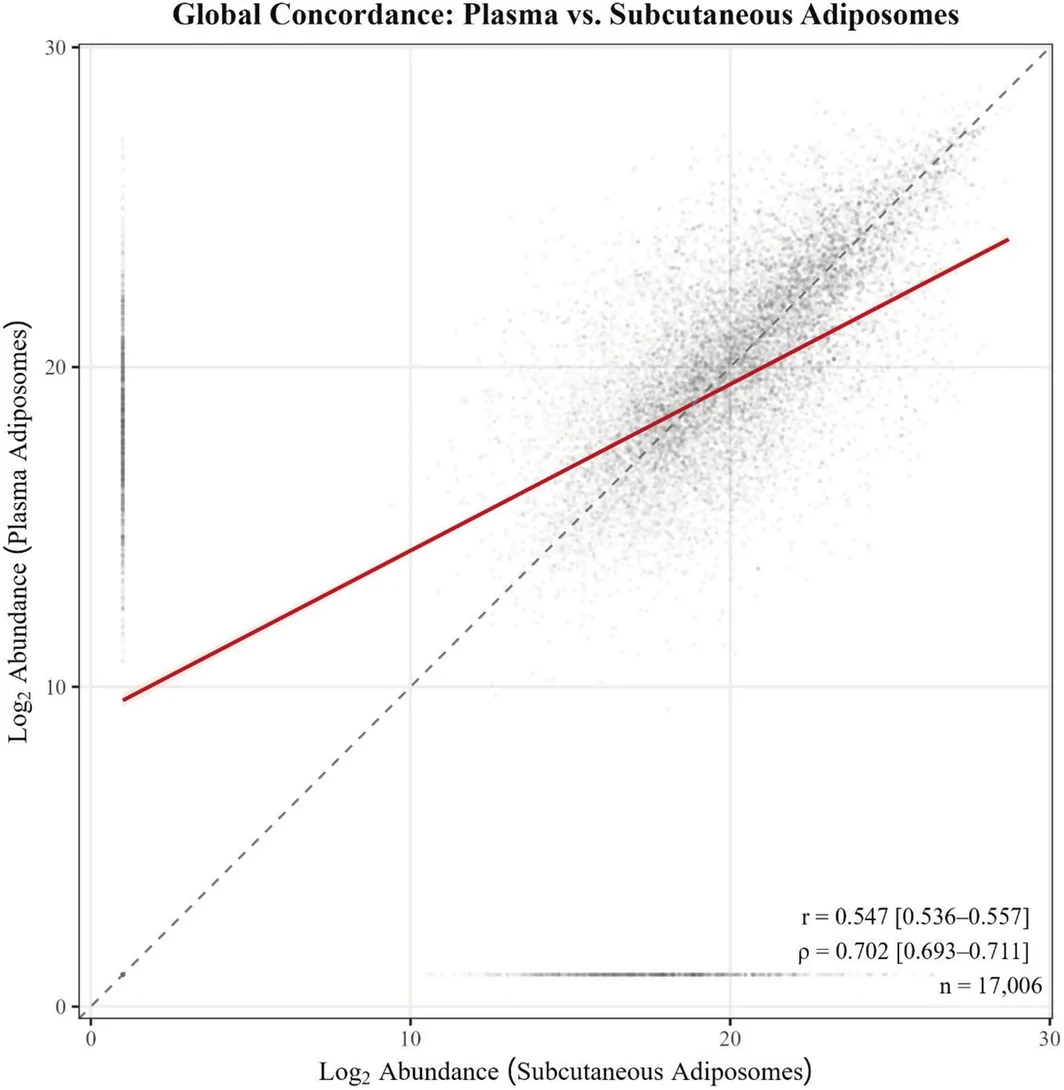
Global concordance between plasma and subcutaneous adiposome lipidomes. Each point represents one lipid feature in one subject (*n* = 17,006 observations). The red line indicates the linear regression fit with 95% confidence band; the dashed gray line represents the line of identity (*y* = *x*). Pearson *r* = 0.547; Spearman *ρ* = 0.702.

### 3.2 Lipid Class Composition

The 18‐class composition of the adiposome lipidome was nearly identical between compartments (Spearman *ρ* = 0.996, *p* < 0.001; Pearson *r* = 0.996 on log_2_‐transformed class percentages; Figure 2). Triacylglycerols dominated both compartments, accounting for 48.0% of the total lipid signal in subcutaneous adiposomes versus 43.3% in plasma, followed by phosphatidylcholines at 15.7% versus 17.8% and sphingomyelins at 12.9% versus 13.3%. Class‐level abundance and rank order were therefore strongly preserved, despite modest proportional differences in selected classes, including ceramides and lysophosphatidylcholines.

**FIGURE 2:**
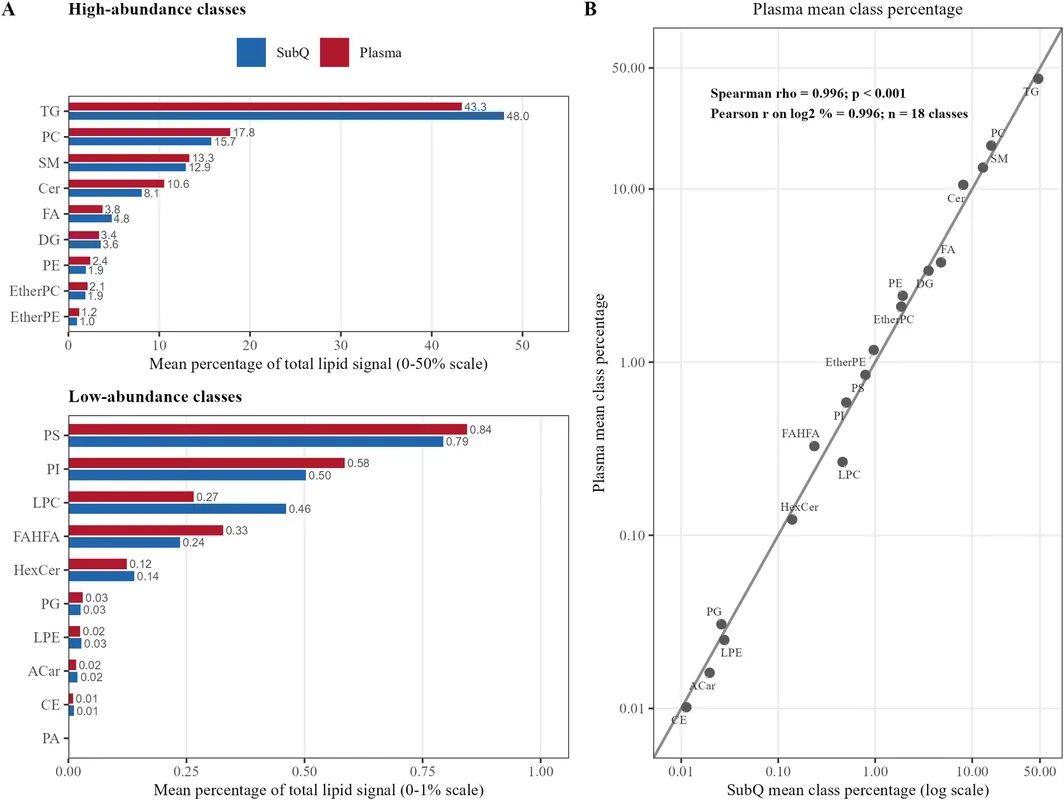
Broad lipid‐class composition profile. Panel (A) shows the mean percentage of total lipid signal for high‐ and low‐abundance broad classes. Panel (B) compares positive class percentages on log‐scaled axes. PA had no detected feature and was excluded from the log‐scale correlation.

### 3.3 Per‐Subject Concordance

Within‐individual concordance varied considerably across the 22 participants. The median Pearson *r* was 0.782 (IQR 0.682–0.848; range 0.225–0.890), and the median Spearman *ρ* was 0.818 (IQR 0.758–0.832; Figure 3). Three participants had Pearson *r* below the prespecified 0.5 threshold (0.231, 0.292, and 0.225), although their Spearman *ρ* values remained above 0.5. Despite this high within‐profile similarity, matched pairs were not more similar than mismatched plasma‐subcutaneous pairs (median *ρ* 0.818 vs. 0.811; Mann–Whitney *p* = 0.620), and the matched sample was the closest cross‐compartment profile for 0 of 22 participants.

**FIGURE 3:**
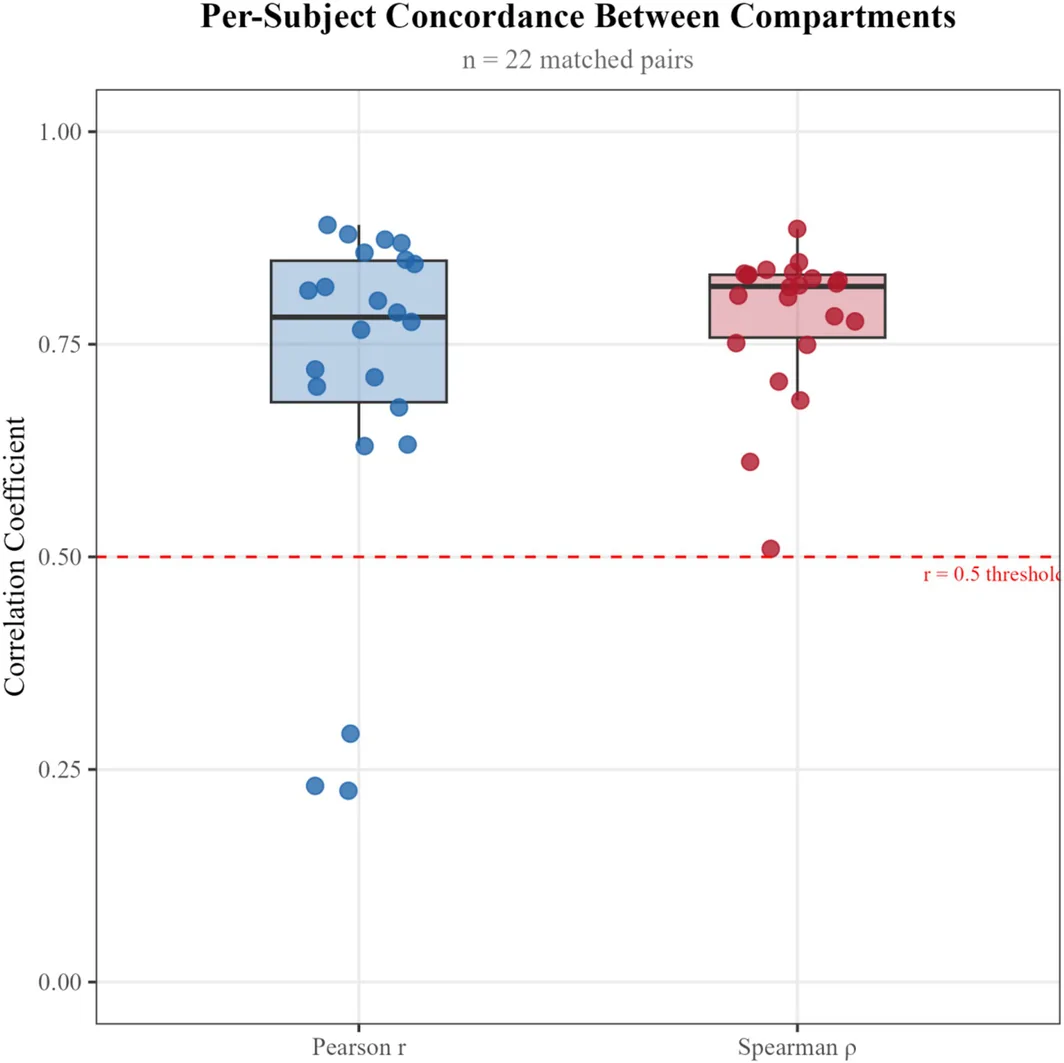
Per‐subject concordance between matched plasma and subcutaneous adiposome lipidomes. Each dot represents one subject (*n* = 22). The red dashed line indicates the *r* = 0.5 threshold used to identify outlier subjects.

### 3.4 Sex and Cardiometabolic Phenotypes

Median matched‐profile *ρ* was 0.807 among women and 0.831 among men (rank‐biserial effect = −0.474; permutation *p* = 0.228; BH *q* = 0.342); the three men were insufficient for reliable sex‐specific inference. None of diabetes, insulin resistance, hypertension, dyslipidemia, hepatic steatosis, systemic inflammation, vascular‐function classification, or FMD classification was associated with matched‐profile similarity after correction (minimum *q* = 0.342; Table 2). Among 23 continuous variables, arteriolar FID was nominally associated with matched‐profile similarity (*ρ* = 0.441; permutation *p* = 0.039), but the association did not remain significant after BH correction (*q* = 0.700; Table 3).

**TABLE 2:** Dichotomous clinical‐phenotype associations with matched whole‐profile similarity.

Phenotype	*n* positive	*n* negative	Median *ρ* positive	Median *ρ* negative	Rank‐biserial effect	Permutation *p*	BH *q*
Sex: female vs. male	19	3	0.807	0.831	−0.474	0.228	0.342
Diabetes	8	14	0.813	0.821	0.000	1.000	1.000
Insulin resistance	9	13	0.807	0.825	−0.094	0.743	0.956
Hypertension	11	11	0.825	0.783	0.339	0.197	0.342
Dyslipidemia	12	10	0.826	0.792	0.317	0.225	0.342
Hepatic steatosis	16	6	0.823	0.763	0.458	0.116	0.342
Systemic inflammation	12	10	0.792	0.829	−0.483	0.059	0.342
Vascular‐function classification: impaired vs. normal	15	7	0.821	0.777	0.352	0.210	0.342
FMD classification: impaired vs. normal	18	4	0.820	0.797	0.056	0.903	1.000

**TABLE 3:** Continuous clinical‐variable associations with matched whole‐profile similarity.

Variable	*n*	Spearman *ρ*	*p*	*q*
Age	22	0.172	0.443	0.942
BMI	22	−0.017	0.942	0.942
Total fat percent	22	−0.274	0.216	0.845
Visceral fat mass	22	−0.140	0.531	0.942
Fasting insulin	22	0.040	0.857	0.942
Fasting glucose	22	0.408	0.061	0.700
HOMA‐IR	22	0.124	0.578	0.942
HbA1C	22	−0.148	0.507	0.942
Total cholesterol	22	0.362	0.095	0.726
LDL	22	0.041	0.854	0.942
HDL	22	0.071	0.753	0.942
Triglycerides	22	0.233	0.294	0.845
Nitric oxide	22	−0.103	0.649	0.942
AST enzyme	22	−0.100	0.650	0.942
ALT enzyme	22	−0.035	0.878	0.942
Protein	22	−0.118	0.603	0.942
Albumin	22	0.090	0.685	0.942
Brachial FMD %	22	0.249	0.262	0.845
Arteriolar FID	22	0.441	0.039	0.700
Leptin	22	−0.043	0.849	0.942
Adiponectin	22	−0.306	0.164	0.845
IL6	22	−0.265	0.229	0.845
CRP	22	−0.018	0.939	0.942

### 3.5 Feature‐Level Concordance and Discordance

Of 773 lipid species, 770 had valid across‐subject plasma‐subcutaneous Spearman estimates. The median *ρ* was 0.101 and the mean *ρ* was 0.089; no species survived Benjamini‐Hochberg FDR correction. Nevertheless, 553 of 770 valid species (71.8%) had positive *ρ* values, exceeding the 50% null expectation (exact binomial *p* = 6.10 × 10^−35^). In the complementary prespecified Pearson‐threshold analysis, 49 features (6.3%) were classified as concordant (*r* > 0.3), 55 (7.1%) as discordant (*r* < −0.2), and 669 (86.5%) as neutral (Figure 4). PE 16:0_22:5, DG 18:1_20:4, and ACar 26:0 were the leading positive Pearson correlations (*r* = 0.957, 0.594, and 0.568, respectively). The most negative Pearson correlations were Cer_AP t42:1 (*r* = −0.530), FA 22:4 (*r* = −0.484), and EtherPC 42:4e (*r* = −0.441). Supplementary Tables 1 and 2 provide the corresponding ranked species results.

**FIGURE 4:**
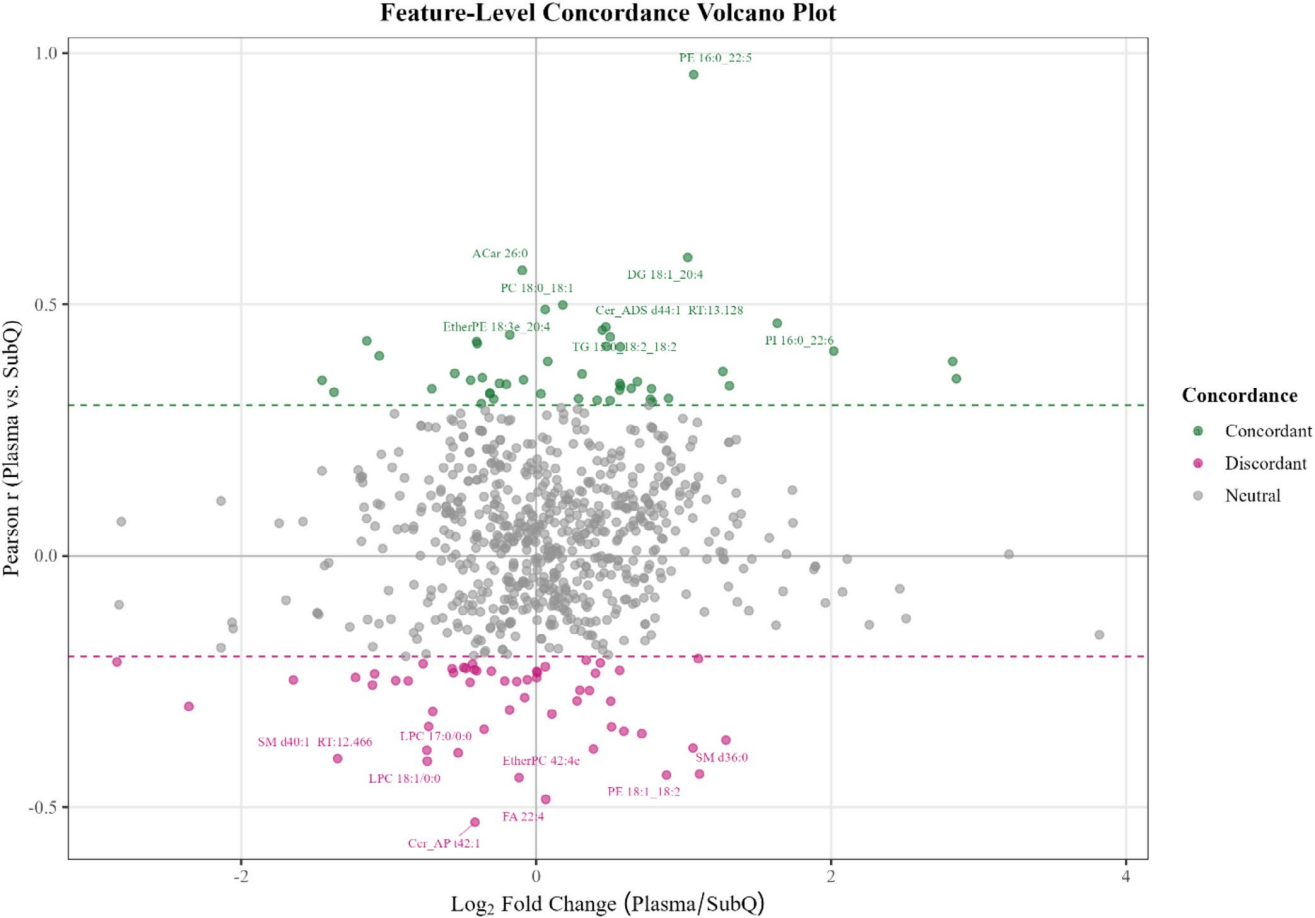
Concordance volcano plot. Each point represents one lipid feature (*n* = 773). The *y*‐axis shows the Pearson correlation between plasma and subcutaneous adiposome abundances across 22 paired subjects. The *x*‐axis shows the log_2_ fold‐change (plasma/subcutaneous). Green: Concordant (*r* > 0.3); pink: Discordant (*r* < −0.2); gray: Neutral.

### 3.6 Multivariate Analysis

Principal component analysis of the Pareto‐scaled lipidomic matrix revealed that the first two principal components accounted for 46.3% and 5.8% of the total variance, respectively (cumulative: 52.1%; Figure 5). Visual inspection of the principal component analysis (PCA) scores plot showed partial overlap between plasma and subcutaneous adiposome clusters, with 95% confidence ellipses intersecting. A permutation‐based test of compartment separation did not reach statistical significance (*p* = 0.181; 1000 permutations), indicating that the two compartments do not form robustly separable multivariate clusters. This result is consistent with the study's central hypothesis: plasma adiposomes partially, but not fully, recapitulate the tissue‐of‐origin lipidome, resulting in overlapping multivariate profiles.

**FIGURE 5:**
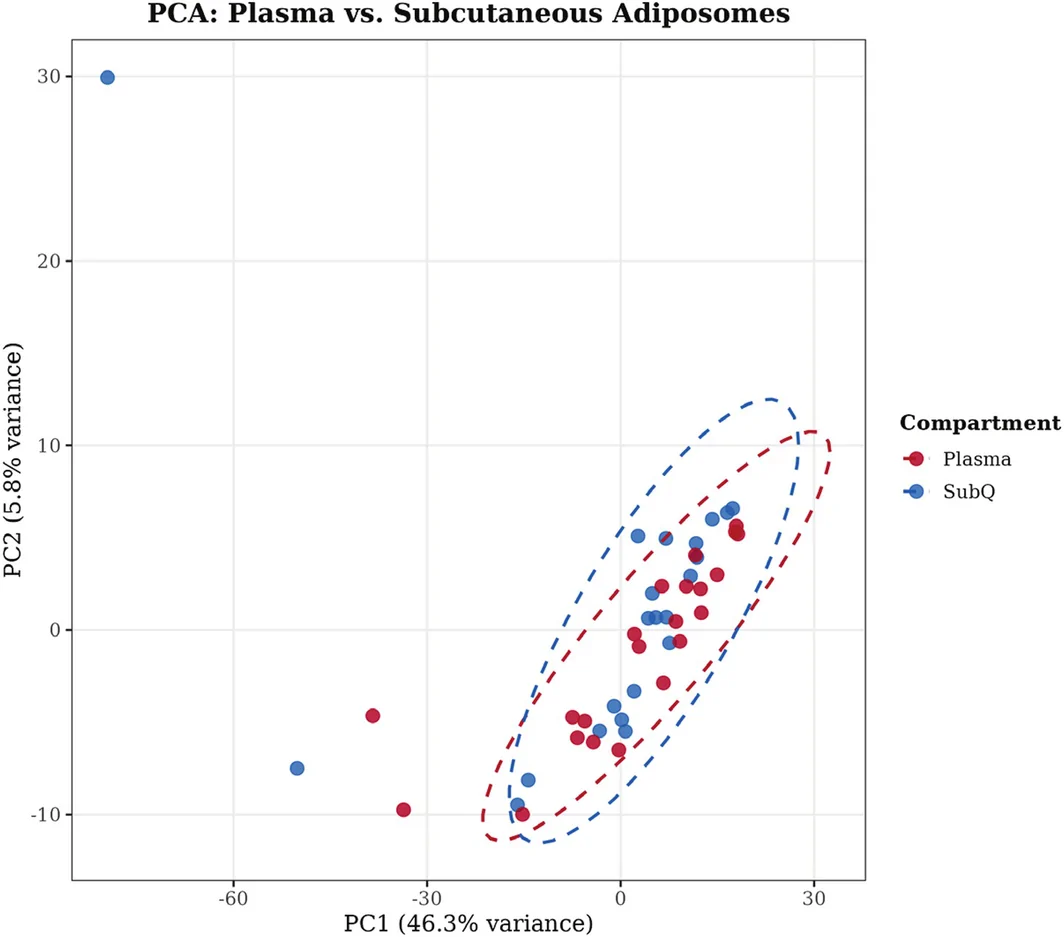
PCA scores plot showing the first two principal components (PC1: 46.3%; PC2: 5.8%). Blue: Subcutaneous adiposomes; red: Plasma adiposomes. Dashed ellipses represent 95% confidence regions.

### 3.7 Bland–Altman Agreement Analysis

The Bland–Altman analysis revealed a small positive mean bias of 0.366 log_2_ units (plasma minus subcutaneous), indicating a modest systematic tendency for plasma adiposome lipid abundance to be slightly higher than subcutaneous values on the log_2_ scale (Figure 6). The 95% limits of agreement were wide (−11.63 to +12.36 log_2_ units), reflecting substantial feature‐level and subject‐level variability. Linear regression of the difference on the mean revealed significant proportional bias (slope = −0.064; *p* < 0.001), indicating that for features with higher mean abundance, plasma and subcutaneous values tend to converge, while low‐abundance features exhibit greater discrepancy. Lipid class‐specific Bland–Altman bias estimates and limits of agreement are presented in Supplementary Table 3.

**FIGURE 6:**
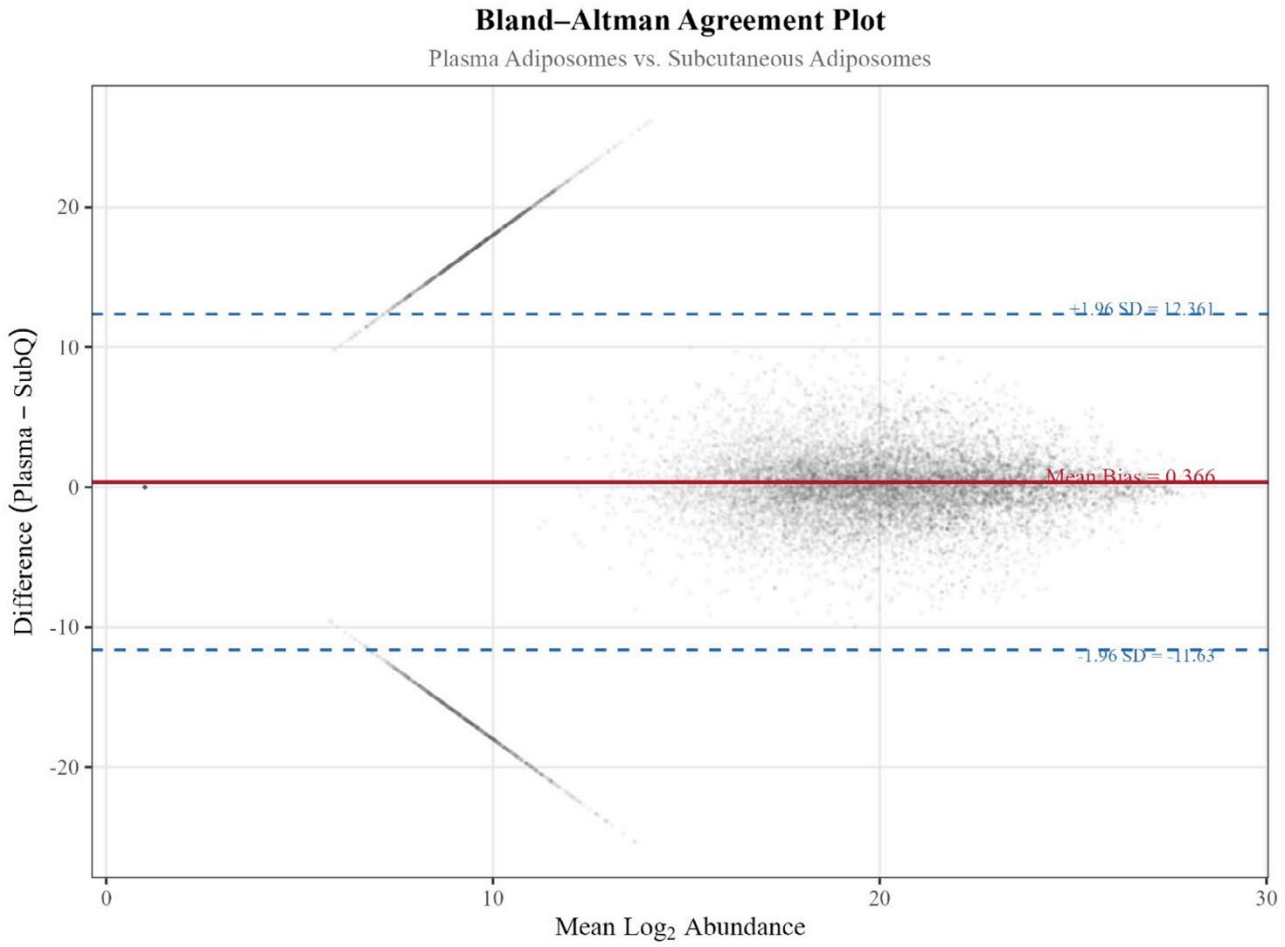
Bland–Altman agreement plot. Each point represents one lipid feature in one subject (*n* = 17,006 observations). The solid red line indicates the mean bias; dashed blue lines indicate the 95% limits of agreement (±1.96 SD).

### 3.8 Lipid Class Enrichment Analysis

Fisher's exact tests did not reveal statistically significant enrichment of any lipid class among concordant or discordant features after Benjamini–Hochberg correction (all *q* > 0.05; Supplementary Figure 1). Neutral features predominated across all lipid classes, particularly TG (146 of 162), PC (126 of 146), Cer (85 of 92), and SM (83 of 94). Among non‐neutral features, LPC was exclusively discordant (5 discordant, 0 concordant; OR = 0; adjusted *p* = 0.527), while SM showed the strongest non‐significant skew toward discordance (9 discordant, 2 concordant; OR = 0.22; adjusted *p* = 0.527). Conversely, PE (4 concordant, 1 discordant; OR = 4.80; adjusted *p* = 0.975) and EtherPE (5 concordant, 2 discordant; OR = 3.01; adjusted *p* = 0.975) tended toward concordance. These exploratory class‐specific patterns require confirmation in larger cohorts.

### 3.9 Differential Abundance Analysis

Paired Wilcoxon signed‐rank tests were performed for each of the 773 lipid features. No individual feature reached the Benjamini‐Hochberg‐adjusted significance threshold. Because the cohort contained 22 matched pairs and 773 simultaneous tests, exploratory fold‐change rankings were not interpreted as validated differential features.

### 3.10 Hierarchical Clustering

Unsupervised hierarchical clustering (Ward's method, Euclidean distance) of the top 100 most variable lipid features produced a heatmap that partially segregated samples by compartment (Supplementary Figure 2). Several subclusters were enriched for either plasma or subcutaneous adiposome samples, although complete separation was not achieved. The variable features were mainly phosphatidylcholines, triglycerides, and sphingomyelins, supporting partial rather than complete preservation of adipose lipid profiles in circulation.

### 3.11 Cross‐Compartment Clinical Associations

Cross‐compartment agreement of species‐level lipid‐clinical effect profiles was modest and selective. Agreement was strongest for sex (effect‐size *ρ* = 0.363; sign concordance 65.5%), fasting insulin (*ρ* = 0.325; 71.6%), HOMA‐IR (*ρ* = 0.308; 73.2%), and age (*ρ* = 0.221; 80.1%; Figure 7), whereas AST, HbA1c, FMD category, and fasting glucose showed discordant profiles. Five plasma broad classes were negatively associated with serum total protein after class‐level correction: Cer, LPC, SM, LPE, and PS; matched subcutaneous associations were not significant. Plasma species‐level ALT and AST signals (265 and 236 species, respectively) collapsed to zero after excluding the participant with extreme liver enzyme values.

**FIGURE 7:**
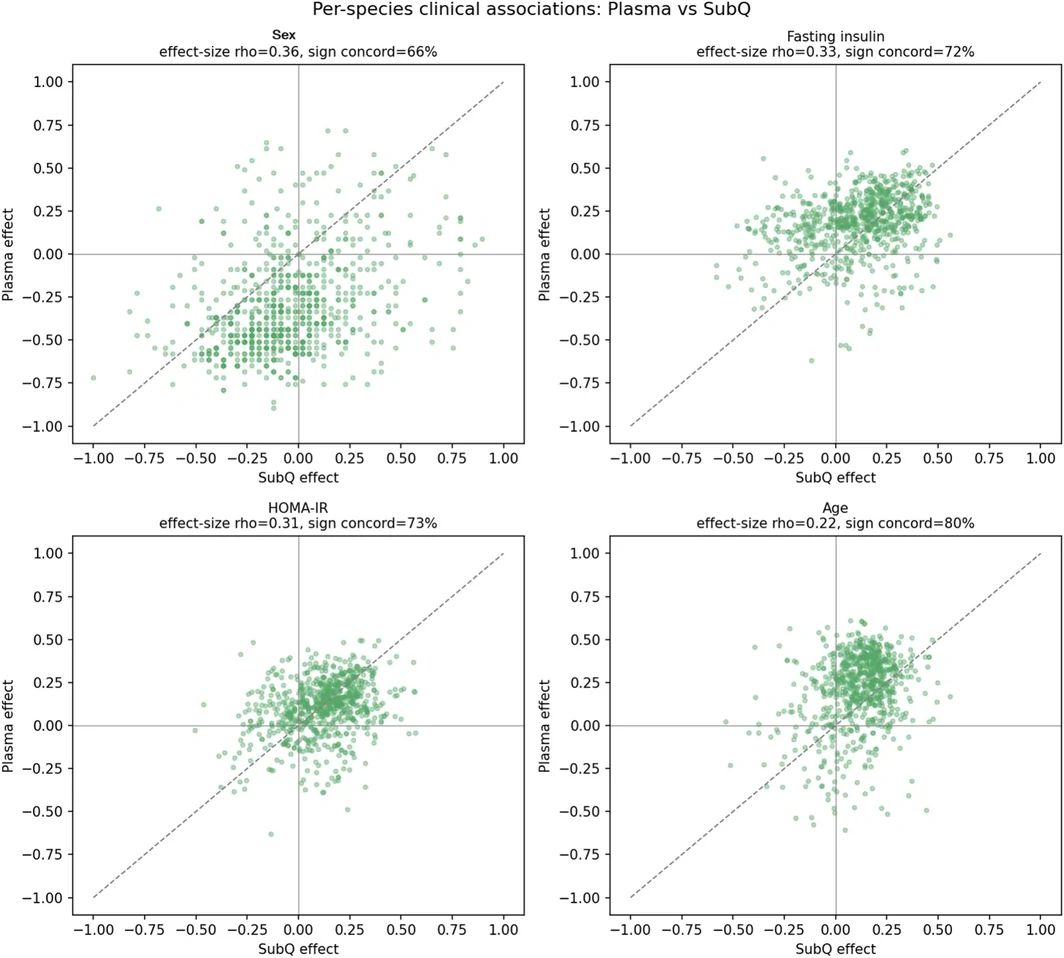
Per‐species cross‐compartment agreement of clinical associations. For each clinical variable, each point is one lipid species, plotted by its association effect size in subcutaneous (*x*‐axis) versus plasma (*y*‐axis) adiposomes. Panels show the four variables with the strongest agreement; the dashed line is the line of identity. Per‐panel values give the effect‐size Spearman *ρ* and the sign concordance.

### 3.12 Focused Species‐Clinical Associations

The focused matrix tested 12 leading species against nine continuous clinical variables in each compartment (Figure 8). Clinical association heatmaps by broad lipid class are presented in Supplementary Figure 3. Two of 216 coefficients were nominally significant: plasma ACar 26:0 with triglycerides (*ρ* = −0.458; *p* = 0.032; full‐screen *q* = 0.988) and Subcutaneous EtherPE 18:1e_20:3 with C‐reactive protein (*ρ* = 0.537; *p* = 0.0099; full‐screen *q* = 0.110). No focused relationship survived correction in the full 773‐species screen, including PE 16:0_22:5 and DG 18:1_20:4.

**FIGURE 8:**
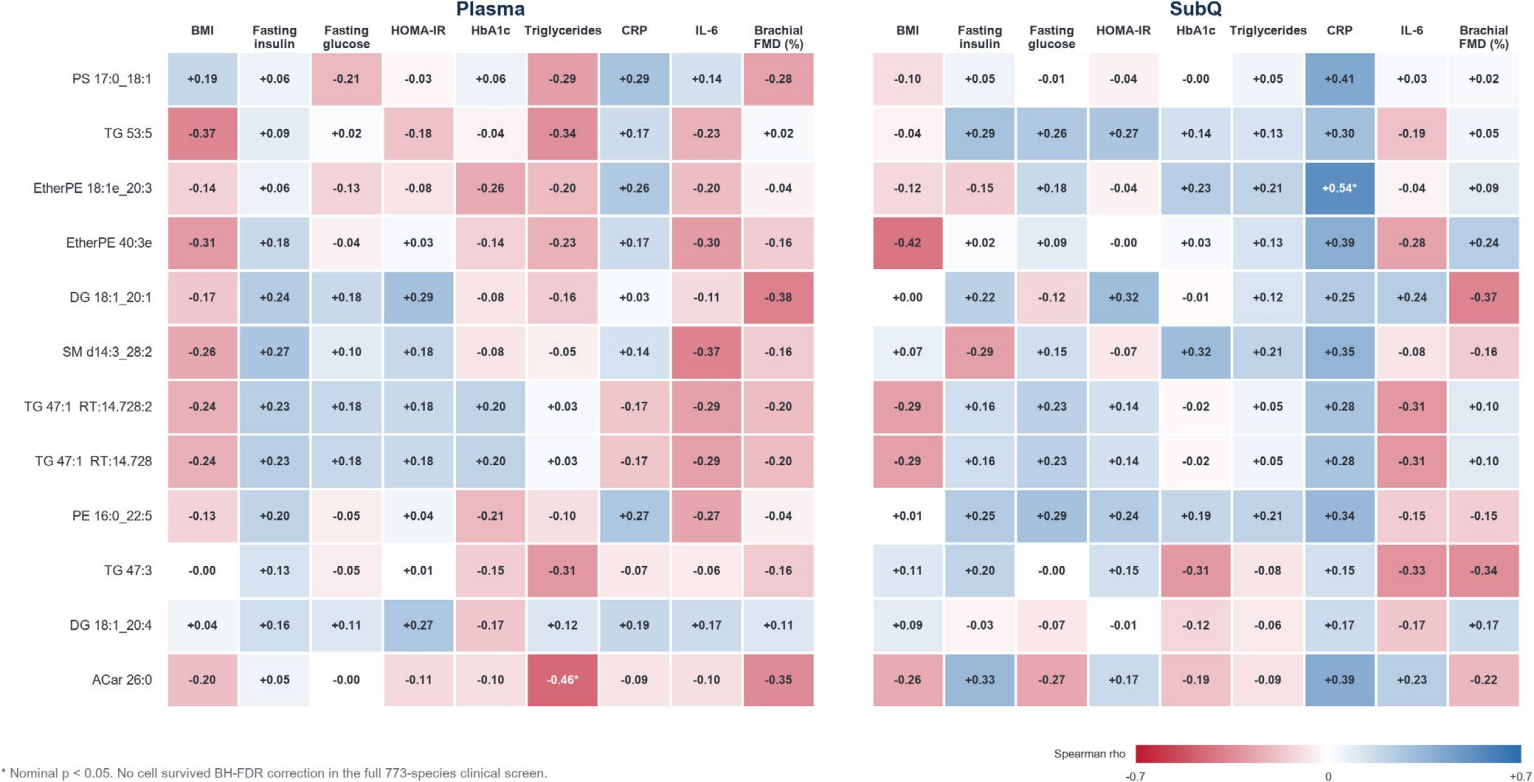
Focused species‐clinical Spearman matrices. Plasma and Subcutaneous panels show *ρ* for 12 leading species across nine prespecified outcomes (*n* = 22). No cell survived Benjamini‐Hochberg FDR correction in the full species‐level clinical screen.

### 3.13 Sensitivity Analyses

Exclusion of the three outlier subjects (016, 018, and 021) improved global concordance from Pearson *r* = 0.547 to *r* = 0.597 (a 9.1% relative increase) and from Spearman *ρ* = 0.702 to *ρ* = 0.759 (an 8.1% relative increase), confirming that these three subjects contributed disproportionately to the attenuation of the overall concordance estimate.

Threshold sensitivity analysis demonstrated that the number of concordant and discordant features was sensitive to the chosen cutoffs (Supplementary Table 4). Under the strict threshold (*r* > 0.4/*r* < −0.3), only 16 features (2.1%) were classified as concordant and 20 (2.6%) as discordant. Under the lenient threshold (*r* > 0.2/*r* < −0.1), these numbers increased to 124 (16.0%) concordant and 150 (19.4%) discordant. Across all threshold levels, concordant and discordant counts were broadly similar, and most lipid features remained neutral. The principal concordance and agreement metrics comparing plasma and subcutaneous adiposome lipidomes are summarized in Table 4.

**TABLE 4:** Summary of key concordance metrics.

Metric	Value
*N* subjects (matched pairs)	22
*N* lipid features	773
*N* broad‐class labels	19 provided; 18 detected (PA absent)
Global Pearson *r* (95% CI)	0.547 (0.536–0.557)
Global Spearman *ρ* (95% CI)	0.702 (0.693–0.711)
Per‐subject Pearson *r*, median [IQR]	0.782 [0.682–0.848]
Per‐subject Spearman *ρ*, median [IQR]	0.818 [0.758–0.832]
Outlier subjects (Pearson *r* < 0.5)	3 (subjects 016, 018, 021)
Pearson‐threshold concordant features (*r* > 0.3)	49 (6.3%)
Pearson‐threshold discordant features (*r* < −0.2)	55 (7.1%)
Neutral features	669 (86.5%)
Significant features (Wilcoxon, *q* < 0.05)	0
PCA: PC1 variance explained	46.3%
PCA: PC2 variance explained	5.8%
Permutation test (compartment separation)	*p* = 0.181
Bland–Altman mean bias	0.366 log_2_ units
Bland–Altman 95% limits of agreement	−11.63 to +12.36
Proportional bias (slope; *p*)	−0.064; *p* < 0.001
Global *r* after outlier exclusion	0.597
Global *ρ* after outlier exclusion	0.759
Valid across‐subject species *ρ* estimates	770
Median across‐species Spearman *ρ*	0.101
Positive species *ρ*	553/770 (71.8%); exact *p* = 6.10 × 10^−35^
Species significant after BH‐FDR	0
Mean class‐composition Spearman *ρ*	0.996
Mean class‐composition Pearson *r* on log_2_%	0.996
Matched versus mismatched median whole‐profile *ρ*	0.818 versus 0.811; *p* = 0.620
Matched pair was closest match	0/22
Women versus men median matched *ρ*	0.807 versus 0.831; *p* = 0.228; *q* = 0.342
Binary phenotype associations after BH‐FDR	0/9
Continuous phenotype associations after BH‐FDR	0/23
Focused species‐clinical associations after BH‐FDR	0/216
Plasma total‐protein broad‐class associations	5 classes at *q* < 0.05
ALT plasma species hits before/after leave‐one‐out	265/0
AST plasma species hits before/after leave‐one‐out	236/0

## 4 Discussion

In this cross‐sectional within‐subject lipidomic analysis, circulating plasma adiposomes partially preserved the global and class‐level lipidomic structure of matched subcutaneous adipose tissue adiposomes, but individual‐level and species‐level fidelity was modest. The pooled correlation is informative about shared architecture but is not a standalone fidelity estimate, because it can conflate between‐class abundance spread with true cross‐compartment agreement. Per‐participant concordance was generally high, but matched profiles were not more similar than mismatched profiles, and no participant's matched sample was the closest cross‐compartment profile. At the broad‐class level, mean composition was highly aligned. PCA showed overlapping compartment profiles, and Bland–Altman analysis retained wide limits of agreement with proportional bias. These results support preservation of broad lipidomic architecture, not quantitative or subject‐specific interchangeability.

This pattern is consistent with selective lipid sorting during extracellular vesicle formation rather than passive transfer of the source‐tissue lipidome (Blandin et al. 2023). This interpretation also builds directly on previously published adiposome lipidomics studies. For example, in a recent study, adiposomes isolated from human adipose tissue in obesity showed increased levels of ceramides, free fatty acids, acylcarnitines, and diacylglycerols, along with depletion of phosphatidylcholines, lysophosphatidylcholines, sphingomyelins, and potentially protective fatty acyl esters of hydroxy fatty acids (Mahmoud et al. 2025). Specific changes included increased Cer‐NS d18:1/23:0 and ACar 16:0 and reduced PC 18:2/20:4 and FAHFA 18:0/20:2. These alterations were associated with greater adiposity, insulin resistance, systemic inflammation, and impaired vascular function, establishing adiposome lipid cargo as a disease‐responsive molecular signature. In a subsequent study, bariatric surgery led to marked remodeling of the adiposome lipidome, with reductions in ceramides, diacylglycerols, phosphatidic acids, and triglycerides, and increases in phosphatidylcholines, phosphatidylethanolamines, sphingomyelins, and FAHFAs (Metwally et al. 2026). Increases in PC 18:2/20:4, LPC 19:0, and PE 18:0/18:2 were among the prominent postsurgical changes, while SM d40:1, SM d42:4, and PC 17:0/18:2 were associated with greater BMI reduction. The present paired analysis extends prior work by showing that these adipose‐related lipid patterns remain detectable in the circulating adiposome fraction at the global and lipid‐class levels, although individual lipid abundances and subject‐specific profiles are only partially preserved. Circulating adiposomes, therefore, appear to represent a remodeled systemic readout of adipose lipid biology rather than direct copies of tissue‐derived adiposomes. In the context of interorgan communication, this suggests that the adiposome lipid signal available to distant organs through the circulation preserves the broad class architecture of its adipose source but is remodeled at the species level.

This pattern of strong class‐level but weaker species‐level concordance likely reflects the sorting of lipids during vesicle formation. Vesicles do not simply sample the parent membrane at random. Certain lipids, such as cholesterol, sphingomyelin, glycosphingolipids, and phosphatidylserine, are enriched two‐ to threefold over their cells of origin because vesicles assemble at lipid rafts and endosomal microdomains (Skotland et al. 2017). This sorting acts mainly on lipid class and acyl‐chain features rather than on every species equally, which could preserve the broad composition we observed while still allowing individual species to differ between tissue and circulation. Ether‐linked phospholipids are one known source of this species‐level variability, as they are abundant in some vesicle populations and sensitive to peroxisomal and membrane‐trafficking activity (Skotland et al. 2019), consistent with the discordance we noted for EtherPC 42:4e.

PE 16:0_22:5, DG 18:1_20:4, and ACar 26:0 were among the leading positive Pearson correlations across compartments. These annotations are consistent with membrane phospholipid structure, diacylglycerol signaling, and fatty acid handling, all of which are relevant to extracellular vesicle biology and metabolic disease (Erion and Shulman 2010; Primrose and Claypool 2024; Dambrova et al. 2022; Koves et al. 2008). No focused relationship survived correction in the full species screen; plasma ACar 26:0 with triglycerides was nominal, whereas PE 16:0_22:5 and DG 18:1_20:4 had no FDR‐significant clinical association. These species should therefore be viewed as hypothesis‐generating signals requiring replication.

The discordant features also require calibrated interpretation. Cer_AP t42:1, FA 22:4, and EtherPC 42:4e had the strongest negative correlations. These species fall within lipid families involved in sphingolipid biology, polyunsaturated fatty acid metabolism, and ether‐linked phospholipid structure (Wang et al. 2021; Gonzalez‐Plascencia et al. 2026; Hu et al. 2025; Klingenberg et al. 2025; Wu et al. 2025). Their discordance may reflect compartment‐specific lipid processing rather than analytical failure, but the present study cannot determine whether these differences arise before vesicle release, during circulation, or during isolation. Class enrichment analysis also remained nonsignificant after correction, even though lysophosphatidylcholines appeared only among discordant features and sphingomyelins skewed toward discordance. Thus, the data support selected class‐level trends for follow‐up, not firm class‐specific conclusions.

The skew of sphingomyelins toward discordance, along with a ceramide species (Cer_AP t42:1) ranking among the most negatively correlated features, is worth noting given the central role of sphingolipids in cardiometabolic disease. Ceramides and related sphingolipids impair insulin signaling, promote endothelial and mitochondrial dysfunction, and accumulate in obesity and dyslipidemia, and they now serve as independent predictors of cardiovascular risk (Chaurasia and Summers 2021). If these bioactive species are generated, converted, or cleared differently once vesicles enter the circulation, their plasma levels may carry distinct or complementary information rather than mirror the tissue pool. This supports our view that structural membrane lipids tend to track between compartments, whereas bioactive sphingolipids and inflammatory species are more prone to remodeling.

Matched self‐plasma and subcutaneous adiposome pairs were not more similar than mismatched pairs, with median self Spearman *ρ* = 0.818 versus mismatch *ρ* = 0.811, *p* = 0.620. This indicates that the observed concordance largely reflects a generic shared lipidome structure rather than subject‐specific tissue‐to‐plasma fidelity. In practical terms, plasma adiposomes should not be treated as personalized circulating copies of each subject's adipose tissue lipidome. Their more defensible use case is in population level, class level, or composite lipid signatures and in longitudinal monitoring, rather than individualized tissue inference from a single plasma sample.

Cross‐compartment agreement of species‐level clinical‐effect profiles was selective and moderate. The strongest agreement was seen for sex, fasting insulin, HOMA‐IR, and age, whereas several hepatic, glycemic, and vascular measures showed discordant patterns. Direct matched‐profile analyses did not identify sex, diabetes, insulin resistance, hypertension, dyslipidemia, hepatic steatosis, systemic inflammation, vascular impairment, or FMD classification as an FDR‐significant explanation of the principal finding. This should not be interpreted as evidence of no effect: only three men were included, phenotypes overlapped, and *n* = 22 limits the stability of subgroup or multivariable inference.

The clinical applicability of these findings therefore lies in candidate biomarker development, risk stratification, and longitudinal monitoring, especially for obesity, diabetes, fatty liver disease, and cardiovascular risk, conditions with substantial health care burden (Lal et al. 2012; Nagi et al. 2025; Kazi et al. 2024). Adipose tissue biopsy is invasive and poorly suited for repeated sampling, whereas plasma adiposome lipidomics is more feasible for serial assessment. However, the present data do not support the use of plasma adiposomes as direct one‐to‐one surrogates for subcutaneous adipose tissue adiposomes. A more realistic translational path would be a targeted plasma adiposome lipid panel derived from reproducible composite patterns, with external validation, standardized isolation, isotope‐labeled standards, and predefined analytic thresholds (Anh et al. 2024). Such panels should be designed to monitor adipose‐related metabolic state, not to reconstruct the full tissue adiposome lipidome.

The biomarker rationale for circulating adiposomes is further supported by evidence that adipose tissue is a major, physiologically active source of cargo for circulating extracellular vesicles. Adipose‐derived vesicles deliver functional cargo to distant organs and can regulate gene expression in the liver and other tissues, leading to their description as a previously unrecognized form of adipokine (Thomou et al. 2017). From this perspective, the plasma adiposome lipidome is biologically meaningful as an integrated, systemically relevant readout of adipose secretory activity, even when it does not completely mirror the tissue‐compartment species‐for‐species. This framing supports the use of composite circulating lipid signatures for metabolic phenotyping while remaining consistent with our finding that individualized tissue inference from a single plasma sample is not yet warranted.

### 4.1 Limitations

Several limitations should be acknowledged. The modest, predominantly female cohort reflected the available regional bariatric surgery population rather than sex‐specific eligibility criteria. Although our concordance analysis did not differ significantly by sex, only three men were included, limiting sex‐specific inference. Cohort heterogeneity and the cross‐sectional design also limited subgroup analyses and assessment of temporal or causal lipidomic changes. EV abundance was normalized to protein content (μg) rather than particle number, followed by baseline correction and normalization of LC–MS signals to internal‐standard signal. Thus, concordance analyses were based on protein‐normalized, internally standardized relative intensities rather than absolute lipid quantities.

Circulating adiposomes may originate from multiple adipose depots, including subcutaneous, visceral, and mesenteric fat. Subcutaneous adipose tissue, which accounts for the majority of total body fat mass, is likely the major contributor to the circulating adiposome pool. Nevertheless, visceral adipose tissue, despite its smaller mass, may exert disproportionate metabolic effects through portal drainage and direct hepatic exposure. Experimental portal drainage of adipose tissue promotes hepatic insulin resistance (Rytka et al. 2011), and human visceral adipose extracellular vesicles can alter inflammatory and metabolic signaling in hepatocytes (Lago‐Baameiro et al. 2025). Thus, the contribution of visceral and mesenteric adipose tissue to the circulating EV lipidome may be functionally significant beyond what their relative mass alone would suggest.

### 4.2 Future Studies and Clinical Implications

Future studies should validate these findings in larger, sex‐balanced, and more diverse cohorts with detailed phenotyping of adiposity, insulin resistance, liver fat, inflammation, vascular function, and cardiovascular outcomes. Further depot‐specific analyses comparing plasma with subcutaneous, visceral, and mesenteric adiposome profiles will be important for delineating the relative contributions of these depots to the circulating adiposome pool and their roles in obesity‐associated metabolic dysfunction. Adipose‐derived extracellular vesicles are increasingly viewed as mediators of communication between adipose tissue and distant organs, including liver, skeletal muscle, vasculature, pancreas, immune cells, and brain (Le Lay and Scherer 2025). Linking circulating adiposome lipid cargo to its uptake and functional effects in these recipient organs will be needed to define its role in interorgan communication. Longitudinal studies before and after weight loss, exercise, bariatric surgery, GLP‐1 receptor agonist therapy, SGLT2 inhibition, or dietary interventions would clarify whether plasma adiposome lipids track therapeutic improvement. Clinical translation will also require standardized targeted assays with isotope‐labeled standards, external quality control, and clinically interpretable thresholds (Anh et al. 2024).

The broader implication is that plasma adiposome lipidomics may become a clinically useful window into adipose tissue dysfunction, provided it is interpreted as a partial and remodeled readout rather than a direct tissue replica. Structural membrane lipids, such as selected phosphatidylethanolamines, may preserve tissue concordance, while bioactive inflammatory, oxidative, and sphingolipid species may show stronger compartment‐specific remodeling. This model is compatible with the current view that adipose extracellular vesicles contribute to metabolic dysregulation and interorgan communication in obesity (Malaguarnera et al. 2025). It also explains why plasma adiposomes could be valuable for clinical risk signatures, even when individual lipid species do not perfectly match those in tissue adiposomes.

## 5 Conclusion

Our paired analysis shows that plasma adiposomes partially reflect the lipidomic profile of matched subcutaneous adipose tissue adiposomes, with preservation of mean broad‐class architecture supporting their potential as accessible circulating readouts of adipose lipid biology and of the lipid cargo that adipose tissue exports for interorgan communication. Although species‐level and subject‐specific concordance was limited, these findings establish a foundation for developing circulating adiposomes as a minimally invasive liquid‐biopsy approach for assessing adipose metabolic state. Future studies using larger cohorts, standardized extracellular‐vesicle workflows, longitudinal sampling, and targeted lipid panels are warranted to evaluate their utility for biomarker development, risk stratification, and therapeutic monitoring.

## Author Contributions

**Mohamed Saad Rakab:** conceptualization, methodology, formal analysis, data curation, visualization, writing – original draft, writing – review and editing. **Imaduddin Mirza:** methodology, investigation, data curation, writing – review and editing. **Elsayed Metwally:** visualization, data curation, writing – review and editing. **Mohammed Morsy:** investigation, data curation, writing – review and editing. **Carlos Martinez:** data curation, formal analysis, writing – review and editing. **Francesco M. Bianco:** investigation, resources, writing – review and editing. **Mario Masrur:** investigation, resources, writing – review and editing. **Chandra Hassan:** investigation, resources, writing – review and editing. **Brian T. Layden:** conceptualization, resources, supervision, writing – review and editing. **Abeer M. Mahmoud:** conceptualization, methodology, resources, supervision, project administration, funding acquisition, writing – review and editing. All authors read and approved the final manuscript.

## Funding

This project was funded by the National Institutes of Health grant number R01HL161386 (PI: Abeer M. Mahmoud).

## Ethics Statement

The study was conducted in accordance with the Declaration of Helsinki and approved by the Institutional Review Board of the University of Illinois at Chicago (protocol #2021‐1113; approval date October 25, 2021).

## Consent

Written informed consent was obtained from all participants before enrollment.

## Conflicts of Interest

The authors declare no conflicts of interest.

## Supporting information


**Supplementary Figure 1:** Lipid class distribution by concordance group.
**Supplementary Figure 2:** Hierarchical clustering heatmap of the top 100 most variable lipid features across 44 samples.
**Supplementary Figure 3:** Clinical association heatmaps by broad lipid class.
**Supplementary Table 1:** Top 10 positive cross‐compartment correlations.
**Supplementary Table 2:** Top 10 negative cross‐compartment correlations.
**Supplementary Table 3:** Bland–Altman bias and limits of agreement stratified by lipid class (top 10 by absolute bias).
**Supplementary Table 4:** Threshold sensitivity analysis for concordance classification.

## Data Availability

The datasets analyzed during the current study are available from the corresponding author upon reasonable request.
