# Supplementary material for: Circulating Plasma Adiposomes as a Partial Lipidomic Readout of Matched Subcutaneous Adipose Tissue: Implications for Interorgan Communication
Source: Compr Physiol. 2026 Nov 1;16(6):e70296. doi: 10.1002/cph4.70296 (PMC13724754; doi:10.1002/cph4.70296)
Supplement: Supplementary file 1 [file CPH4-16-e70296-s001.docx]

**Supplementary Table 1. Top 10 positive cross-compartment correlations.**

| **Feature** | **Pearson r** | **Spearman ρ** | **Log₂FC** |
| --- | --- | --- | --- |
| PE 16:0_22:5 | 0.957 | 0.516 | 1.07 |
| DG 18:1_20:4 | 0.594 | 0.397 | 1.03 |
| ACar 26:0 | 0.568 | 0.473 | −0.09 |
| PC 18:0_18:1 | 0.499 | 0.109 | 0.18 |
| EtherPE 18:3e_20:4 | 0.490 | 0.319 | 0.06 |
| PI 16:0_22:6 | 0.463 | 0.436 | 1.63 |
| Cer_ADS d44:1 (RT 13.128) | 0.455 | 0.465 | 0.47 |
| TG 15:0_18:2_18:2 | 0.449 | 0.435 | 0.45 |
| DG 16:0_18:3 | 0.440 | 0.265 | −0.18 |
| EtherPE 18:1e_20:3 | 0.435 | 0.552 | 0.50 |

Exploratory rankings; no species survived FDR correction

**Supplementary Table 2. Top 10 negative cross-compartment correlations.**

| **Feature** | **Pearson r** | **Spearman ρ** | **Log₂FC** |
| --- | --- | --- | --- |
| Cer_AP t42:1 | −0.530 | −0.389 | −0.41 |
| FA 22:4 | −0.484 | −0.298 | 0.06 |
| EtherPC 42:4e | −0.441 | −0.394 | −0.12 |
| PE 18:1_18:2 | −0.436 | −0.401 | 0.88 |
| SM d36:0 | −0.434 | −0.329 | 1.11 |
| LPC 18:1/0:0 | −0.408 | −0.023 | −0.74 |
| SM d40:1 (RT 12.466) | −0.403 | −0.237 | −1.35 |
| LPC 17:0/0:0 | −0.392 | −0.461 | −0.53 |
| OxPC 16:0_18:3+1O | −0.386 | −0.463 | −0.74 |
| PC 16:0_20:3 (RT 10.351) | −0.384 | −0.251 | 0.39 |

Exploratory rankings; no species survived FDR correction

**Supplementary Table 3. Bland–Altman bias and limits of agreement stratified by lipid class (top 10 by absolute bias).**

| **Lipid Class** | **n** | **Mean Bias** | **SD** | **Lower LoA** | **Upper LoA** |
| --- | --- | --- | --- | --- | --- |
| LPC | 616 | -1.212 | 8.20 | -17.29 | 14.87 |
| PE | 770 | 1.184 | 6.06 | -10.69 | 13.05 |
| PI | 374 | 1.124 | 5.01 | -8.69 | 10.93 |
| EtherPE | 572 | 0.996 | 6.62 | -11.98 | 13.97 |
| PG | 198 | 0.694 | 8.76 | -16.47 | 17.85 |
| Cer | 2024 | 0.678 | 3.68 | -6.54 | 7.89 |
| PC | 3234 | 0.591 | 7.09 | -13.31 | 14.49 |
| SM | 2090 | 0.365 | 6.25 | -11.89 | 12.62 |
| EtherPC | 880 | 0.291 | 5.79 | -11.05 | 11.64 |
| ACar | 132 | -0.252 | 7.91 | -15.76 | 15.26 |

**Supplementary Table 4. Threshold sensitivity analysis for concordance classification.**

| **Threshold** | **Concordance** | **Discordance** | **N Concordant** | **N Discordant** |
| --- | --- | --- | --- | --- |
| Strict | r > 0.4 | r < −0.3 | 16 | 20 |
| Default | r > 0.3 | r < −0.2 | 49 | 55 |
| Lenient | r > 0.2 | r < −0.1 | 124 | 150 |

**Supplementary Figure 1.** Lipid class distribution by concordance group. Bubble size reflects the number of lipid features in each class-concordance category.

**Supplementary Figure 2.** Hierarchical clustering heatmap of the top 100 most variable lipid features across 44 samples. Columns are annotated by compartment (blue: subcutaneous; red: plasma). Rows are annotated by lipid class. Clustering was performed using Ward’s method with Euclidean distance.

**Supplementary Figure 3.** Clinical association heatmaps by broad lipid class. Cells show class-total Spearman rho after all detected features were reassigned to the provided broad labels. PA is shown as NA because no detected feature mapped to that label.
